# Niche Differentiation of Bacterial Versus Archaeal Soil Nitrifiers Induced by Ammonium Inhibition Along a Management Gradient

**DOI:** 10.3389/fmicb.2020.568588

**Published:** 2020-11-12

**Authors:** Di Liang, Yang Ouyang, Lisa Tiemann, G. Philip Robertson

**Affiliations:** ^1^Department of Plant, Soil and Microbial Sciences and Great Lakes Bioenergy Research Center, Michigan State University, East Lansing, MI, United States; ^2^W. K. Kellogg Biological Station, Michigan State University, Hickory Corners, MI, United States

**Keywords:** niche differentiation, long-term, management gradient, ammonia oxidizing archaea, ammonia oxidizing bacteria, resistance, archaea, nitrification inhibition

## Abstract

Soil nitrification, mediated mainly by ammonia oxidizing archaea (AOA) and bacteria (AOB), converts ammonium (NH_4_^+^) to nitrite (NO_2_^−^) and thence nitrate (NO_3_^−^). To better understand ecological differences between AOA and AOB, we investigated the nitrification kinetics of AOA and AOB under eight replicated cropped and unmanaged ecosystems (including two fertilized natural systems) along a long-term management intensity gradient in the upper U.S. Midwest. For five of eight ecosystems, AOB but not AOA exhibited Haldane kinetics (inhibited by high NH_4_^+^ additions), especially in perennial and successional systems. In contrast, AOA predominantly exhibited Michaelis-Menten kinetics, suggesting greater resistance to high nitrogen inputs than AOB. These responses suggest the potential for NH_4_^+^-induced niche differentiation between AOA and AOB. Additionally, long-term fertilization significantly enhanced maximum nitrification rates (*V_max_*) in the early successional systems for both AOA and AOB, but not in the deciduous forest systems. This was likely due to pH suppression of nitrification in the acidic forest soils, corroborated by a positive correlation of *V_max_* with soil pH but not with *amoA* gene abundance. Results also demonstrated that soil nitrification potentials were relatively stable, as there were no seasonal differences. Overall, results suggest that (1) NH_4_^+^ inhibition of AOB but not AOA could be another factor contributing to niche differentiation between AOA and AOB in soil, and (2) nitrification by both AOA and AOB can be significantly promoted by long-term nitrogen inputs.

## Introduction

Soil nitrification is the microbial process that oxidizes ammonia (NH_3_) into nitrite (NO_2_^−^) and nitrate (NO_3_^−^), and is central to the global nitrogen cycle as it influences ecosystem nitrogen retention (Kowalchuk and Stephen, 2001) and can regulate the forms of reactive N in the environment (Robertson and Groffman, 2015). Unlike NH_4_^+^, which binds with cation-exchange sites on soil organic matter and mineral surfaces, both NO_2_^−^ and NO_3_^−^ are mobile anions, and can thereby be easily leached from soils to result in water pollution (Robertson and Vitousek, 2009). In addition, nitrification is an important biological process leading to emissions of N_2_O (Davidson et al., 1986), a potent greenhouse gas with a global warming potential ~300 times higher than CO_2_. N_2_O also destroys stratospheric ozone (Ravishankara et al., 2009). As NH_4_^+^ can enter ecosystems *via* multiple processes including soil organic matter mineralization, N_2_ fixation, deposition, and fertilizer application, understanding the factors regulating soil nitrification is important for improving subsequent N use efficiencies and for reducing the negative environmental impacts of reactive N.

For over 100 years, ammonia oxidizing bacteria (AOB), first cultured in 1890 (Frankland and Frankland, 1890), were believed to be the sole agents of autotrophic nitrification. This view was transformed by the discovery of the ammonia oxidizing archaea (AOA) *Nitrosopumilus maritimus* SCM1 in 2005 (Könneke et al., 2005). Since then the numerical dominance of AOA over AOB in diverse ecosystems, including terrestrial (Leininger et al., 2006), marine (Wuchter et al., 2006), and hot springs (Hatzenpichler et al., 2008), have been widely reported. In addition, because *N maritimus* SCM1 exhibits reduced nitrification activity with NH_4_^+^ additions ≥2 mm and has a much higher substrate affinity than most AOB (Martens-Habbena et al., 2009), NH_4_^+^ affinity has been often considered an important factor leading to niche differentiation between AOA and AOB. However, the substrate affinity of terrestrial AOA can be similar to that of some AOB (Kits et al., 2017).

The view that AOA prefer oligotrophic environments while AOB dominate nutrient-enriched environments (Schleper, 2010) has been challenged by the discovery of soil AOA “*Ca. Nitrosocosmicus franklandus*,” which can tolerate NH_4_^+^ at higher concentrations than most AOB strains (Lehtovirta-Morley et al., 2016). This finding also revealed the lack of knowledge about niche differentiation induced by NH_4_^+^ inhibition and the overlooked role AOA may play in fertilized soils. It seems clear that our understanding of NH_4_^+^ sensitivity in soil AOA is constrained by the limited numbers of pure cultures and enrichments available (Jung et al., 2011, 2014; Lehtovirta-Morley et al., 2011, 2014, 2016; Tourna et al., 2011). In addition, as phylogenetic studies have revealed an extensive diversity of soil nitrifiers, it is unknown if the existing cultures and enrichments of AOA and AOB are environmentally representative (Prosser and Nicol, 2012).

To eliminate the inherent biases of isolation techniques and directly measure the responses of *in situ* nitrifier communities to NH_4_^+^ additions, NH_4_^+^ tolerance can also be investigated *via* nitrification kinetics (Norton and Stark, 2011). Several studies have examined the effects of NH_4_^+^ additions on soil nitrification in general (Lu et al., 2015; Mushinski et al., 2017), but the potentially different responses of AOA and AOB to gradients of NH_4_^+^ have not yet been well articulated (Ouyang et al., 2017). Existing soil nitrification kinetic studies have mainly focused on a single management type and without differentiating between AOA and AOB (Stark and Firestone, 1996; Koper et al., 2010; Auyeung et al., 2015). Thus, it is unclear how kinetics of AOA and AOB might be differentially affected in ecosystems with varying management intensities.

Here we investigate the nitrification kinetics of AOA and AOB separately along a long-term management intensity gradient in the upper U.S. Midwest. We use the selective inhibitor 1-octyne (Taylor et al., 2013, 2015), which inhibits the AOB ammonia monooxygenase (AMO), to separate AOA from AOB nitrification in whole soil. We selected ecosystems ranging from intensively managed annual row crops to unmanaged late successional deciduous forest (including long-term N fertilizer treatments in successional systems), which allowed us to test the hypotheses that (1) management intensities differentially affect nitrification by AOA and AOB; (2) both AOA and AOB nitrification positively respond to long-term N fertilization; (3) high soil NH_4_^+^ levels can lead to niche differentiation between AOA and AOB, and (4) environmental variables such as soil pH can be a positive predictor shaping nitrification kinetic parameters for both AOA and AOB.

## Materials and Methods

### Study Site

This study was conducted in the Main Cropping System Experiment (MCSE) of the Kellogg Biological Station (KBS) LTER site located in southwest Michigan (42° 24'N, 85° 23'W). The MCSE was established in 1988 and includes 11 ecosystems that form a management intensity gradient on the same soil series: annual crops, perennial crops, and unmanaged systems at different stages of ecological succession (Robertson and Hamilton, 2015). The KBS climate is humid continental with 1,005 mm annual precipitation spread evenly throughout the year and a 10.1°C mean annual temperature (30-year mean from 1981). Soils are well drained Alfisol loams (Typic Hapludalfs of co-mingled Kalamazoo and Oshtemo series), formed from glacial till and outwash with some intermixed loess (Crum and Collins, 1995; Luehmann et al., 2016). Average sand and clay contents in surface soils are 43 and 17%, respectively (Robertson and Hamilton, 2015).

We studied three types of systems: (1) two annual cropping systems, one conventionally managed corn-soybean-winter wheat rotation (Conventional) and the other similar but biologically managed (Biologically-based); (2) a hybrid poplar system (Poplar); and (3) three successional systems of different ages: an early successional system (Early successional), a never-tilled annually mown grassland system (Grassland), and a late successional deciduous forest (Deciduous forest). The two annual cropping systems and the Poplar and Early successional systems are replicated as 1 ha plots (90 × 110 m) in each of six randomized blocks; four were selected for this study. The Grassland system is replicated four times and the Deciduous forest system is replicated three times at nearby locations on the same soil series (Robertson and Hamilton, 2015). In addition, subplots receiving long-term N fertilizer addition in the Early successional (Fertilized Early successional; since 1990) and the Deciduous forest (Fertilized Deciduous forest; since 2007) were also sampled.

The Conventional system has received standard rates of N fertilizer since establishment in 1988: 137 ± 20 kg N ha^−1^ year^−1^ for corn and 77 ± 17 kg N ha^−1^ year^−1^ for wheat (Gelfand et al., 2016). Soybeans received 0–5 kg N ha^−1^ year^1^. N fertilizer was mostly applied as urea-ammonium nitrate (28-0-0). The Biologically-based system has received no N fertilizer; instead, winter cover crops, including red clover (*Trifolium pratense* L.) during the wheat phase prior to corn, and rye grass (*Lolium multiflorum* L.) following corn harvest before soybean, provide additional N. Red clover is a legume that fixes N_2_ and both red clover and ryegrass scavenge soil N otherwise leached or denitrified during non-crop seasons. Tillage for both systems included chisel plowing followed by secondary tillage. Herbicides were used to suppress weeds in the Conventional system and additional tillage provided weed control in the Biologically-based system.

The Poplar system was planted in 1989 to *Populus* × *canadensis* Moench “Eugenei.” Fertilizer was applied as 123 kg N ha^−1^ ammonium nitrate in the establishment year and the first harvest was in 1999. After the second harvest in 2008 and 1 fallow year, *Populus nigra* × *Populus maximowiczii* “NM6” was planted in 2009. Fertilizer was then applied once in 2011 at 157 kg N ha^−1^ as ammonium nitrate.

The Early successional system was abandoned from agriculture in 1989 and has been burned every spring since 1997 to exclude woody plants. Canada goldenrod (*Solidago canadensis* L.), Kentucky bluegrass (*Poa pratensis* L.), arrow leaved aster (*Aster sagittifolius*), and timothy grass (*Phleum pratense* L.) were the dominants during the current study.^1^ Since 1990, a 5 × 5 m subplot located at the northwest corner of each of the Early successional plots was fertilized annually with 120 kg N ha^−1^ ammonium nitrate pellets in early July (Huberty et al., 1998; Grman et al., 2010). The Grassland system was established on a cleared woodlot ca. 1959 and has never been plowed. Grass is mown annually to inhibit woody species. Current dominants include smooth brome grass (*Bromus inermis* Leyss.), Canada goldenrod (*S canadensis* L.), tall oatgrass (*Arrhenatherum elatius* L.), blackberry (*Rubus allegheniensis* Porter), sassafras (*Sassafras albidum*), and Kentucky bluegrass (*Poa pratensis* L.).^2^ The late successional Deciduous forest stands are unmanaged and have never been plowed. Overstory dominants include red oak (*Quercus rubra* L.), pignut hickory (*Carya glabra* Mill.), white oak (*Quercus. alba* L.) and sugar maple (*Acer saccharum* Marsh.).^3^ Since 2007, a 2 × 2 m subplot in each of the Deciduous forest stands was fertilized annually with 100 kg N ha^−1^ year^−1^ ammonium nitrate or urea applied in three doses per year during the growing season as 4-L solutions.

### Soil Sampling

Soils were sampled seasonally for testing nitrification potentials, soil pH, and *amoA* gene abundance and once for nitrification kinetics. For testing nitrification potentials, soil pH, and *amoA* gene abundance, soils from all systems but the Grassland were sampled in summer (late June 2016), winter (early December 2016), and spring (early May 2017). Grassland soils were sampled when determining nitrification kinetics, for which samples were collected in 2017 from all systems in the period of late September to early December, after first having established no seasonal patterns for nitrification potentials. For all experiments, five random soil samples per plot (0–15 cm depth) were composited by plot and then passed through a 4 mm mesh sieve immediately on return to the lab. About 15 g of sieved soil were then transferred to a −80°C freezer for future DNA extraction and the remaining soil was stored at 4°C before analysis, which occurred within 4 days.

### Nitrification Potential and Soil pH

To evaluate seasonal patterns of nitrification potentials, 5 g of fresh sieved soil were placed in 155 ml Wheaton bottles amended with 50 ml deionized water containing 10 mM NH_4_Cl. We used 1-octyne, a recently developed and tested chemical inhibitor specific to AOB to distinguish the relative contribution of AOA and AOB (Taylor et al., 2013, 2015). We used a gradient of octyne concentrations ranging from 0–10 μM aqueous concentration (C_aq_) to test for optimal inhibition and we found 4 μM C_aq_ sufficient to inhibit AOB nitrification activities in all soils (Supplementary Figure 1A), which is in agreement with previous studies (Taylor et al., 2013). We also found no evidence of heterotrophic nitrification as 10 Pa of C_2_H_2_ could completely inhibit nitrification activity in our soils (Hynes and Knowles, 1982; Supplementary Figure 1B).

We chose to use 10 mM NH_4_^+^ to investigate the seasonal pattern of nitrification potentials because in a separate study (Liang, 2019), we found this concentration maximizes nitrification-derived N_2_O emissions in most of our soils. Although 10 mM of NH_4_^+^ addition will inhibit some AOB activities as shown later in our nitrification kinetics experiment, we deem this an inconsequential problem for evaluating seasonal trends in potential nitrification rates.

Capped bottles with or without 4 μM C_aq_ octyne were immediately placed on a shaker table and shaken for 24 h at a constant speed of 200 rpm at room temperature (25°C). Samples for NO_2_^−^ + NO_3_^−^ and NH_4_^+^ were taken at 2 and 24 h and nitrification rates were calculated as NO_2_^−^ + NO_3_^−^ accumulations over 22 h. Slurry pH was buffered naturally as no pH change was detected during the incubation. Since it is not entirely clear if the complete ammonia oxidizers (“comammox”) *Nitrospira* are sensitive or resistant to octyne, we cannot rule out the possibility of comammox in contributing to nitrification in our soils (Daims et al., 2015; van Kessel et al., 2015; Li et al., 2019a). Nevertheless, recent evidence seems to suggest only a minor role for comammox in soil NH_3_ oxidation (Wang et al., 2020). Thus, we attribute nitrification (NO_2_^−^ + NO_3_^−^ production) in the presence of octyne to AOA. Nitrification from AOB is calculated as the difference between total nitrification (without octyne) minus AOA nitrification. NO_2_^−^ + NO_3_^−^ and NH_4_^+^ were measured by a Lachat QuikChem 8,500 flow injection analyzer (Hach, Loveland, CO).

To test soil pH, we placed 15 g of sieved soil into extraction cups containing 30 ml deionized water; cups were subsequently capped and shaken to form slurries. We then removed the caps and let slurries stand for at least 30 min before measuring soil pH. Soil moisture was determined by oven drying sieved soil at 60°C for 48 h until constant mass.

### Nitrification Kinetics

For soils from each ecosystem, we placed 5 g of fresh sieved soil into a 155 ml Wheaton bottle. Then we added (NH_4_)_2_SO_4_ to make eight different NH_4_^+^ concentrations ranging from 0.01 to 15.0 mM (0.01, 0.05, 0.1, 0.5, 1, 5, 10 and 15 mM NH_4_^+^) with a final liquid volume of 50 ml. Bottles were capped and placed on a shaker table at a constant speed of 200 rpm at room temperature (25°C) for 24 h. Samples for initial NO_2_^−^ + NO_3_^−^ and NH_4_^+^ were taken after 2 h, at which time we then added either 2.8 mL octyne stock gas (see Taylor et al., 2013 for octyne stock gas preparation) to create a 4 μM C_aq_ concentration, or 2.8 ml of air without octyne. Another set of NO_2_^−^ + NO_3_^−^ and NH_4_^+^ samples were taken at 24 h. Total nitrification (expressed as mg N kg^−1^ dry soil day^−1^) was calculated as NO_2_^−^ + NO_3_^−^ accumulation over 22 h, with AOA nitrification defined as nitrification in the presence of octyne, and AOB nitrification defined as the difference between total nitrification and AOA nitrification. Nitrification kinetics were based on measured NH_4_^+^ concentrations (averaged between 2 and 24 h), so included any NH_4_^+^ produced from net N mineralization during the incubation.

Nitrification kinetics were fit to Michaelis-Menten models using the equation:

$$V=\frac{V_{\max}S}{K_{m}+S}$$ (1)

where, V is the nitrification rate, *V_max_* is the maximum nitrification rate under conditions of unlimited substrate (NH_4_^+^), *S* is the NH_4_^+^ concentration, and *K_m_* is the half-saturation constant, which represents the NH_4_^+^ concentration when the nitrification rate is ½ *V_max_*. *V_max_* reflects the maximum capacity of a soil to oxidize NH_4_^+^, and *K_m_* reflects the NH_4_^+^ affinity of soil ammonia oxidizers. In addition, because nitrification can be inhibited at very high NH_4_^+^ concentrations (Suwa, 1994), we also fitted data with Haldane models when appropriate (Stark and Firestone, 1996; Koper et al., 2010):

$$V=\frac{V_{\max}S}{K_{m}+S+S^{2}/K_{i}\;}$$ (2)

The Haldane model introduces a third parameter *K_i_* that reflects the maximum NH_4_^+^ concentration at which nitrification rates are ½ *V_max_*.

There were three instances where nitrification rates were very low and best-fit Michaelis-Menten models yielded negative values for *K_m_*. In these cases we re-fit the models with *K_m_* constrained to positive values.

### DNA Extraction and Quantification of *amoA* Gene Abundance

Soil DNA was extracted with the Qiagen DNeasy PowerSoil Kit (Qiagen, Germantown, MD) using 0.30 g field-moist soil. The abundance of soil AOB and AOA was quantified by targeting the *amoA* gene, which encodes the alpha subunit of AMO, with primer *amoA*-1F/*amoA*-2R (Rotthauwe et al., 1997) and Arch-amoAF/Arch-amoAR (Francis et al., 2005), respectively. Quantitative PCR was performed on QuantStudio 7 Flex (Genomic Core, Michigan State University) with Power SYBR Green Master Mix (Applied Biosystems, Foster City, CA). The 25 μl reaction mixture including 12.5 μl Power SYBR Green Master Mix, 1.25 μl forward and reverse primers (10 μM), 9 μl molecular grade water, and 1 μl soil DNA extract (10-fold diluted) was first mixed in 96-well plates and then 20 μl aliquots were transferred to a 384-well plate before analysis. The thermal cycling conditions for quantitative PCR were as follows: initial denaturation at 95°C for 10 min, followed by 40 cycles of denaturation at 95°C for 45 s, annealing at 60°C (AOB) or 58°C (AOA) for 1 min and a final extension at 72°C for 45 s. Fluorescence intensity was measured during the 72°C step of each cycle. Standard curves were constructed with plasmids containing cloned *amoA* products from soil DNA from the same site. Amplification efficiencies ranged between 76 and 98%, with standard curve *R*^2^ for both AOA and AOB > 0.99.

### Statistical Analysis

#### ANOVA for Seasonal Nitrification Potentials

Seasonal nitrification potentials were analyzed with PROC GLIMMIX of SAS 9.4 (SAS Institute, Cary, NC, United States). The statistical model included eight ecosystem types × three seasons × two sources of nitrification potentials and interactions among them were considered fixed factors. Field replicates nested within ecosystem types and the interaction between field replicates and seasons nested within ecosystem types were considered random factors. ANOVA was performed by considering ecosystem types as a whole plot factor and season and sources of nitrification as subplot and sub-subplot factors. Homogeneity of variance assumptions were checked by Levene’s test and because AOA and AOB had significantly different residual variability (*p* < 0.05), heterogeneous variance for sources of nitrification potentials was included in the statistical model by a random _residual/group = source statement. Normality of residuals was visually inspected, and no violations of assumptions were detected.

#### Comparison of Kinetic Parameters for AOA and AOB

For model comparisons, we first used the “nls” function in R (version 3.5.0, R Core Team, 2018) to obtain Akaike’s Information Criterion (AIC) values for Michaelis-Menten and Haldane kinetic models for AOA and AOB in every ecosystem. Then an *F*-test was conducted using the “anova” function in R to further determine model superiority. The more complicated Haldane kinetic model was selected (Table 1) only when smaller AIC values and a statistically better fit than for the Michaelis-Menten model were observed (*p* < 0.1).

**Table 1:** Comparisons between Michaelis-Menten and Haldane kinetics models for ammonia oxidizing bacteria (AOB)-derived or ammonia oxidizing archaea (AOA)-derived nitrification rates among different ecosystems; ecosystems followed by “+N” indicate subplots receiving long-term N fertilizer.

Ecosystem	Taxon	Model AIC		Model comparison		Model selection
		Michaelis-Menten	Haldane	*F*	*p*	
Conventional	AOB	90.8	NA	NA	NA	Michaelis-Menten
	AOA	31.1^*^	32.9	0.10	0.76	Michaelis-Menten
Biologically-based	AOB	83.6	82.0^*^	3.45	0.075^**^	Haldane
	AOA	42.4	NA	NA	NA	Michaelis-Menten
Poplar	AOB	64.9	61.6^*^	5.23	0.031^**^	Haldane
	AOA	5.20	5.17^*^	1.88	0.18	Michaelis-Menten
Early successional	AOB	66.3^*^	67.5	0.78	0.38	Michaelis-Menten
	AOA	37.0^*^	38.7	0.29	0.60	Michaelis-Menten
Early successional+N	AOB	93.8	92.7^*^	2.94	0.098^**^	Haldane
	AOA	90.3	NA	NA	NA	Michaelis-Menten
Grassland	AOB	8.79^*^	10.2	0.53	0.47	Michaelis-Menten^***^
	AOA	23.4^*^	24.5	0.77	0.39	Michaelis-Menten^***^
Deciduous forest	AOB	−18.2	−23.2^*^	7.52	0.019^**^	Haldane
	AOA	−12.5	−31.3^*^	3.48	<0.01^**^	Haldane
Deciduous forest+N	AOB	21.0	16.0^*^	7.23	0.016^**^	Haldane
	AOA	14.3^*^	16.2	0.088	0.77	Michaelis-Menten^***^

AIC represents Akaike information criterion. NA (not applicable) appears where there was no ammonium (NH_4_^+^) inhibition observed so a Haldane model could not be fit.

* Model with lower AIC.

** Haldane model provides statistically better fit than Michaelis-Menten model (*p* < 0.1).

*** Required constraining *K_m_* to a positive value as noted in Methods.

Once appropriate models were chosen, we performed a Bayesian *F*-test (Kéry, 2010a) to investigate how management intensities affected kinetic parameters in the managed systems (Conventional, Biologically-based, Poplar, and Grassland), and a Bayesian T-test (Kéry, 2010b) to explore fertilization effects on kinetic parameters for unmanaged systems (Early successional and Deciduous forest; Table 2). We modeled each of the kinetic parameters of different ecosystems following a normal distribution:

$$Parameter_{i}\sim Normal\;\left(\mu_{i},\tau_{i}{}^{2}\right)$$ (3)

$$\mu_{i}\sim Normal\;\left(mean_{i},SD_{i}{}^{2}\right)$$ (4)

**Table 2:** Kinetics parameters of managed systems and unmanaged systems for AOA and AOB nitrification; “+N” indicates subplots receiving long-term N fertilizer.

			AOA			AOB			AOA vs. AOB		
			*V_max_*	*K_m_* (μM)	*K_i_* (mM)	*V_max_*	*K_m_* (μM)	*K_i_* (mM)	*V_max_*	*K_m_*	*K_i_*
Managed	Conventional		1.44 (0.09)^b^	5.67 (4.34)^a^	–	4.80 (0.29)^c^	22.8 (9.6)^b^	–	√	NS	–
	Biologically-based		2.82 (0.11)^c^	2.84 (1.64)^a^	–	5.29 (0.36)^c^	9.18 (3.46)^b^	52.2 (32.1)^a^	√	NS	–
	Poplar		0.78 (0.06)^a^	2.26 (3.51)^a^	–	1.90 (0.27)^b^	6.91 (6.79)^ab^	14.8 (9.5)^a^	√	NS	–
	Grassland		0.86 (0.07)^a^	0.44^**^ (0.37)^a^	–	0.38 (0.05)^a^	0.60^**^ (0.56)^a^	–	√	NS	–
Unmanaged	Early successional	0N	0.89 (0.08)	0.02 (1.86)	–	1.30 (0.14)	4.32 (4.26)	–	√	NS	–
		+N	2.11 (0.21)^*^	9.14 (6.82)	–	3.15 (0.45)^*^	21.5 (12.4)	23.6 (18.2)	√	NS	–
	Deciduous forest	0N	0.67 (0.04)	0.14 (1.46)	12.5 (3.3)	0.57 (0.05)	1.18 (2.42)	24.5 (12.0)	NS	NS	NS
		+N	0.61 (0.08)	0.49^**^ (0.44)	–	0.87 (0.35)	33.6 (37.3)	2.01 (2.38)^*^	NS	NS	–

Parameters are estimated with either Michaelis-Menten or Haldane kinetics models based on the model selection results in Table 1. For each ecosystem, a “–” indicates Michaelis-Menten model is applied and thus *K_i_* does not exist. Numbers within the parentheses represent standard errors. ^a-c^Lower case letters indicate significantly different kinetics parameters among ecosystems (*p* < 0.05). For *K_m_* and *K_i_* values based on ammonia (NH_3_), see Supplementary Table 1. √: kinetics parameters of AOA are significantly different from AOB within the same ecosystem (*p* < 0.05). NS: kinetics parameters of AOA are not significantly different from AOB within the same ecosystem.

* kinetics parameters of +N treatments are significantly different from 0 N (*p* < 0.05).

** *K_m_* values were estimated by constraining “nls” estimates >0.

where, $Parameter_{i}\;$represents *V_max_*, *K_m_* or *K_i_*, and $\mu_{i}$ and $\tau_{i}{}^{2}$ represent mean and variation of the parameter, respectively. $mean_{i}$ and $SD_{i}$ were the estimated kinetic parameters and their standard errors obtained by “nls” function, which were then specified as prior information when we conducted Markov Chain Monte Carlo (MCMC) simulations to sample posterior parameter distributions with JAGS (Plummer, 2003) and the “jagsUI” package for R (Kellner, 2017). We assumed a vague prior for $\tau_{i}{}^{2}$. We ran three chains of 15,000 iterations with 2,000 burn-in iterations with a thinning rate of three, which yielded 13,002 total samples for posterior distribution. All the posterior distributions for kinetic parameters were used for analysis.

#### *amoA* Gene Abundance, Soil pH, and Correlational Analysis

Soil pH and log-transformed *amoA* gene abundance were first averaged across three seasons, which were then correlated with *V_max_* using the “lm” function in R. Constancy of variance and residual normality were checked by plotting residuals against predicted values, with no apparent violation of assumptions observed.

We performed one-way ANOVA to investigate the effects of management intensities on averaged soil pH. In addition, to study how *amoA* gene abundance of AOA and AOB was affected by different ecosystems, a hierarchical model including eight ecosystem types, two taxa, and their interactions was established. We considered field replicates nested within ecosystem types as a random factor. Thus, ecosystem type and taxa were the whole plot and subplot factors, respectively. Statistical analyses were conducted with PROC MIXED and PROC GLIMMIX of SAS 9.4 and heterogeneous variance for ecosystem types was included by a Repeated/group = ecosystem statement based on Levene’s test. Normality of residuals was visually inspected, and no violations of assumptions were detected. Pairwise comparisons among different ecosystems were conducted and we refer to *p* < 0.05 as significantly different throughout the paper.

## Results

### Seasonal Nitrification Potentials

**Figure 1:**
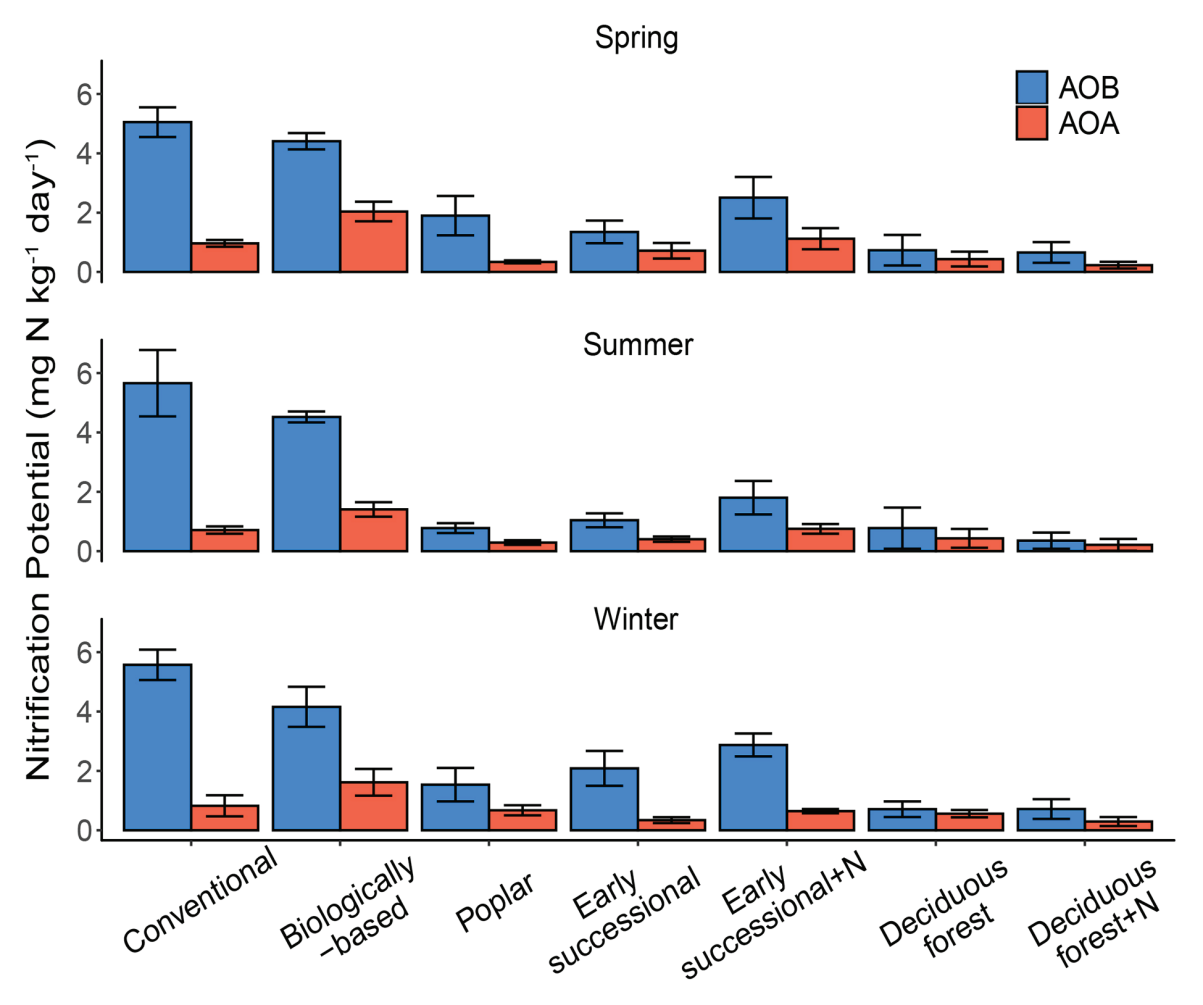
AOA (orange) and AOB (blue)-derived seasonal nitrification potentials in systems varying in management intensities; bars represent standard error (*n* = 4 field replicates except for deciduous forest *n* = 2–3). “+N” indicates subplots receiving N fertilizer. No significant differences among seasons were detected (*p* = 0.25).

Conventional and Biologically-based systems had the highest AOB-derived potential nitrification rates across three seasons, ranging between 4.16 and 5.66 mg N kg^−1^ day^−1^ (Figure 1). In comparison, Deciduous forest and its fertilized subplots were associated with the lowest seasonal AOB-derived nitrification potentials, 0.26–0.51 and 0.27–0.35 mg N kg^−1^ day^−1^, respectively. In general, AOB-derived nitrification potentials in cropping systems were significantly higher than in perennial and successional systems (*p* < 0.05), although Conventional and Biologically-based systems did not significantly differ from each other for two out of three seasons.

For AOA, the Biologically-based system had a seasonal nitrification potential of 1.40–2.04 mg N kg^−1^ day^−1^, which was significantly higher than other systems (*p* < 0.05). No seasonal differences for AOA-derived nitrification potentials were detected among Poplar, Early successional, Deciduous forest nor their fertilized subplots except for the spring sampling period, for which the mean nitrification potential for the Fertilized Early successional system was significantly higher than Poplar and Fertilized Deciduous forest systems (*p* < 0.05). In addition, although not significant, long-term fertilization resulted in 1.4–1.9 times as high as nitrification potentials in Early successional but not in Deciduous forest systems compared with their non-fertilized main plots for both AOA and AOB.

No overall seasonal differences in nitrification potentials were detected (*p* = 0.25). Similarly, there were no detectable two-way interaction effects between season and ecosystem types (*p* = 0.95) or season and nitrifier taxa (*p* = 0.48), nor were there detectable three-way interaction effects among season, ecosystem types, and nitrifier taxa (*p* = 0.50). AOB overall had significantly higher nitrification potentials than AOA (*p* < 0.05); AOB nitrification potentials were consistently higher (*p* < 0.05) than AOA nitrification potentials for Conventional, Biologically-based, and Fertilized Early successional systems in every season but were similar (*p* > 0.05) in Deciduous forest and its fertilized subplots (all three seasons) and in Poplar and Early successional systems (two out of three seasons; Figure 1).

### Kinetics of AOA and AOB

**Figure 2:**
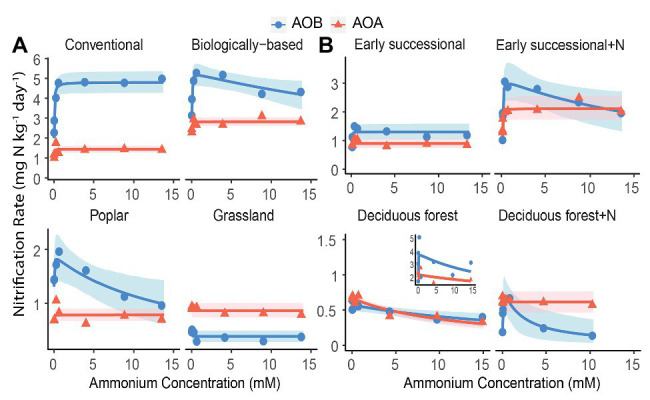
Nitrification kinetics of **(A)** systems varying in management intensities and **(B)** unmanaged systems with (+N) or without long-term N fertilization; Michaelis-Menten or Haldane models were fit to AOB-derived (blue line) and AOA-derived (orange line) nitrification rates. Blue circles and orange triangles are the mean nitrification rates at each ammonium addition. Note y-axis scale differs by systems. Shading represents 95% bootstrap confidence intervals based on *n* = 3–4 field replicates except for Deciduous forest and its fertilized subplots where *n* = 2–3. Inset shows one removed replicate from Deciduous forest adjacent to a dairy farm. Ammonium addition ranged between 0.05 and 15 mM for Poplar and annual cropping systems because NO_3_^−^ + NO_2_^−^ accumulation at 0.01 mM cannot be reliably estimated; ammonium addition ranged between 0.01 and 10 mM for Fertilized Deciduous forest because NO_3_^−^ + NO_2_^−^ accumulation at 15 mM was too low to be detected. For all other systems, ammonium additions ranged between 0.01 and 15 mM.

We conducted an AIC-based model comparison to choose kinetics parameters for AOA and AOB (Table 1). For AOA, Michaelis-Menten kinetic models provided significantly better fits (*p* < 0.1) in all but the Deciduous forest system, whereas for AOB, Haldane kinetic models fitted best (*p* < 0.1) except for Conventional and Grassland systems. Nitrification of AOB in the Early successional system showed slight inhibition at high NH_4_^+^ concentrations (Figure 2B), but overall a Michaelis-Menten kinetic model fitted better (Table 1).

Among managed ecosystems, Conventional and Biologically-based soils were associated with the highest *V_max_* for AOB, reaching 4.80 ± 0.29 (standard error of the mean) and 5.29 ± 0.36 mg N kg^−1^ day^−1^, respectively (Figure 2A). In comparison, significantly lower *V_max_* for AOB were found in Poplar and Grassland systems (*p* < 0.05), 1.90 ± 0.27 and 0.38 ± 0.05 mg N kg^−1^ day^−1^, respectively. In addition, the Conventional system had the highest AOB-derived *K_m_* of 22.8 ± 9.6 μM NH_4_^+^ (Table 2), which was significantly higher than that in Grassland (*p* < 0.05) but not in the Biologically-based or Poplar system. For AOA, *V_max_* was significantly lower than AOB (*p* < 0.05) in all ecosystems except in the Grassland. The highest *V_max_* for AOA was 2.82 ± 0.11 mg N kg^−1^ day^−1^ in Biologically-based soils, which was significantly higher than in all other systems (Figure 2A, *p* < 0.05). Additionally, *V_max_* for AOA in the Conventional system was significantly higher than that in either Poplar or Grassland systems (*p* < 0.05). No significant differences for *K_m_* of AOA were detected among managed ecosystems (Table 2). Moreover, both Biologically-based and Poplar systems exhibited nitrification inhibition for AOB at high NH_4_^+^ concentrations (>1 mM), with *K_i_* of 52.2 ± 32.1 and 14.8 ± 9.5 mM NH_4_^+^, respectively.

Long-term N fertilization resulted in a significant increase (*p* < 0.05) in *V_max_* for both AOA (2.11 ± 0.21 vs. 0.89 ± 0.08 mg N kg^−1^ day^−1^) and AOB (3.15 ± 0.45 vs. 1.30 ± 0.14 mg N kg^−1^ day^−1^) in the Early successional system (Figure 2B); *V_max_* in the fertilized Early successional subplots was about 2.4 times higher than that in the main plots. Similarly, although not significant, *K_m_* for both AOA and AOB was enhanced by long-term fertilization. For the Deciduous forest system, no fertilization-induced increases for *V_max_* and *K_m_* for either AOA or AOB were detected. However, the AOB-derived *K_i_* in the fertilized subplots was significantly lower than that in the unfertilized main plots (*p* < 0.05). In addition, regardless of fertilization, *V_max_* of AOB was significantly higher than AOA in the Early successional (*p* < 0.05) but not in the Deciduous forest system; neither *K_m_* nor *K_i_* were significantly different between AOA and AOB in the Early successional or the Deciduous forest system.

### Soil pH, *amoA* Gene Abundance, and Their Correlations With *V_max_*

**Figure 3:**
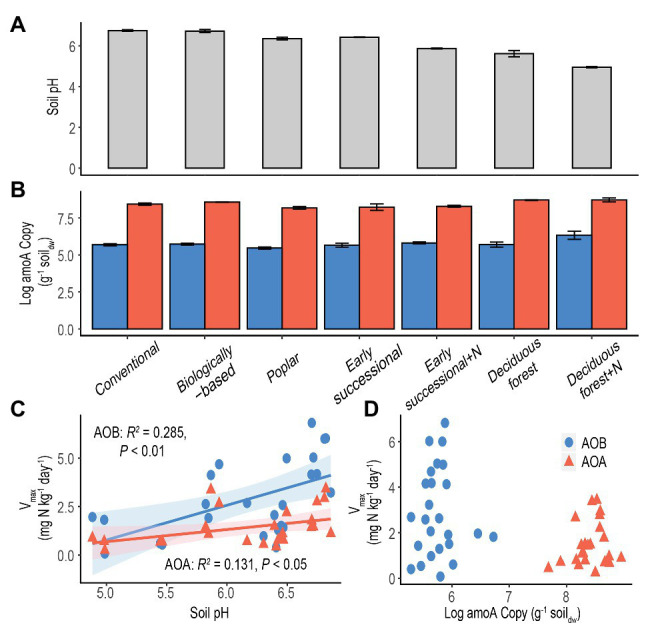
Soil pH **(A)** and log-transformed *amoA* copy numbers **(B)** in systems along a management intensity gradient; “+N” indicates subplots receiving long-term N fertilizer. Bars represent standard errors based on *n* = 4 field replicates except for Deciduous forest and its fertilized subplots where *n* = 3. Correlations between maximum nitrification rate (*V_max_*) with soil pH **(C)** or log-transformed *amoA* gene abundance **(D)** are shown for all systems. A Deciduous forest site adjacent to a dairy farm is not included. Blue dots/bars represent AOB and orange triangles/bars represent AOA. Insignificant value of *p* are not shown for (D).

Conventional and Biologically-based systems had the highest soil pH, 6.74 ± 0.04 and 6.72 ± 0.08, respectively, significantly higher than other systems (*p* < 0.05, Figure 3A). Soil pH was lowest in the Deciduous forest system and its fertilized subplots, 5.61 ± 0.16 and 4.95 ± 0.03, respectively. In addition, long-term fertilization significantly reduced soil pH in both Early successional and Deciduous forest systems (*p* < 0.05).

The abundance of *amoA* genes was significantly higher for AOA than for AOB in each ecosystem (*p* < 0.05, Figure 3B). In addition, for both AOA and AOB, Poplar and Fertilized Deciduous forest systems were associated with the lowest and highest *amoA* gene abundance, respectively. Long-term N fertilization led to significantly more abundant *amoA* genes in Deciduous forest (*p* < 0.05) but not in Early successional systems for both AOA and AOB. Moreover, soil pH was significantly correlated with both *V_max_* for AOA (*p* < 0.05) and AOB (*p* < 0.01) and explained 13.1 and 28.5% of total variance, respectively (Figure 3C). In comparison, no significant relationship was found between *amoA* gene abundance and *V_max_* for AOA or AOB (Figure 3D).

## Discussion

Results show that both nitrification potentials and nitrification kinetics of AOA and AOB responded to management intensities and long-term N fertilization, as summarized below. Results also suggest the potential for niche differentiation between AOA and AOB by NH_4_^+^ inhibition, especially in perennial and successional ecosystems, and as well suggest pH is a strong predictor of nitrification kinetics for both AOA and AOB.

### AOA and AOB Responses to Management Intensity

Consistent with the first hypothesis, management intensities significantly and differentially affected nitrification by both AOA and AOB (*p* < 0.05). For AOB, nitrification potentials in Conventional and Biologically-based systems were significantly higher than in Poplar, Early successional, or Deciduous forest systems for all three seasons (Figure 1, *p* < 0.05). Similarly, *V_max_* of AOB in annual cropping systems was significantly higher than in Poplar and Grassland systems (Figure 2A, Table 2, *p* < 0.05). These results may reflect the differential N input received by various systems. Compared with perennial or successional systems that have never or rarely been fertilized, row crop soils have been receiving chemical N fertilizer (Conventional) or relying on cover crop N (Biologically-based) annually since 1988. Thus, our results are in agreement with previous studies reporting elevated AOB-derived nitrification in fertilized agricultural soils (Taylor et al., 2010, 2012; Zeglin et al., 2011; Ouyang et al., 2016).

For AOA, we found a significantly higher nitrification potential in the Biologically-based agricultural soil (*p* < 0.05) compared with all other systems, but no significant differences were detected among Conventional, Poplar, Early successional and Deciduous forest soils for each season (Figure 1). Similarly, AOA *V_max_* in the Biologically-based system was about 2–3.5 times as high as in other managed systems (*p* < 0.05, Figure 2A, Table 2). These results seem to suggest that cover crops, rather than chemical fertilizers, facilitated AOA nitrification. Previous studies have shown nitrification activities of AOA were stimulated when NH_4_^+^ is mainly derived from organic matter (Gubry-Rangin et al., 2010; Stopnišek et al., 2010; Levičnik-Höfferle et al., 2012; Hink et al., 2017, 2018). Thus, it seems likely that the decomposition of cover crop-derived organic matter promoted AOA nitrification in the Biologically-based system.

In our second hypothesis, we hypothesized that both AOA and AOB positively respond to long-term N fertilization. In the Early successional system, long-term N fertilization resulted in a 40–90% increase in nitrification potentials (Figure 1), and as well a significantly higher *V_max_* in fertilized subplots than in the unfertilized main plots (Figure 2B, Table 2, *p* < 0.05). Positive responses in this system were expected because fertilizer as ammonium nitrate (120 kg N ha^−1^) has been applied annually for over 30 years. Notably, 30 years of continuous fertilizer application in the Early successional system resulted in an even higher *V_max_* for AOA (2.11 ± 0.21 mg N kg^−1^ day^−1^) than in the Conventional system (1.44 ± 0.092 mg N kg^−1^ day^−1^) where chemical fertilizer was only applied in corn and wheat phases. Thus, we present evidence that AOA nitrification activities could be significantly stimulated by long-term inorganic N application, which contrasts with most of the studies reporting either no response (Ouyang et al., 2016, 2017) or negative responses (Giguere et al., 2015). The discrepancy between this study and previous ones could be explained by the slower growth of AOA than AOB under high NH_4_^+^ conditions (Prosser and Nicol, 2012), such that a consistent and long-term fertilization regime (e.g., 30 years) is necessary to observe the positive responses of AOA nitrification. In contrast, the no or negative responses of AOA might reflect the fact that in nitrification potential assays, a single set of lab conditions (e.g., 1 mM of NH_4_^+^ addition) typically create sub-optimal environments that favor AOB nitrification activities over AOA (Hazard et al., 2020).

Contrary to the second hypothesis, no fertilization effects were observed in the Deciduous forest system (Figures 1, 2B). This lack of response might be explained by the low soil pH. Previous research has demonstrated that NH_3_, rather than NH_4_^+^, was the most likely substrate for AMO (Suzuki et al., 1974; Herbold et al., 2017), and NH_3_ concentrations are significantly reduced in acidic environments because NH_3_ is mainly in its protonated form (NH_4_^+^ ⇄ NH_3_, pK_a_ = 9.25). Fertilized Deciduous forest soils had a pH of 4.95 ± 0.03 (Figure 3A), which was significantly lower than its unfertilized counterpart (pH 5.61 ± 0.16, *p* < 0.05) and, as well the Early successional system and its fertilized subplots (pH 5.87–6.42, *p* < 0.05). Despite the low nitrification potentials in Fertilized Deciduous forest soils, these soils had the highest nitrifier abundance: the *amoA* gene for AOB was significantly more abundant here than in all other systems (*p* < 0.05, Figure 3B). Overall, results suggest that low pH inhibited nitrification activities in fertilized forest soils despite the fact that AOB population size was stimulated by the long-term N fertilization. Alternatively, the low soil pH may have selected for an abundance of acidophilic nitrifiers with low nitrification activities.

Results from the seasonal nitrification potential survey also show soil nitrifier communities have been established and nitrification activities were relatively constant after 30 years of consistent management because we found: (1) no significant seasonal differences in nitrification potentials and (2) no significant two-way or three-way interactions effects among season, ecosystem types, and nitrifier taxa (Figure 1). This study therefore provides evidence that for both AOA and AOB, soil nitrification potentials can be very stable across years despite temporal variations in soil temperature, moisture, oxygen, and inorganic N contents (Boone et al., 1999). O’Sullivan et al. (2013) also demonstrated a lack of correlation between total nitrification potential and sampling season in southern Australian agricultural soils but it is unknown if their conclusions apply to AOA and AOB separately.

### The Influences of Management Intensities on *K_m_* of AOA and AOB

The NH_4_^+^ affinities (1/*K_m_*) of AOA were consistently higher than AOB in all ecosystems but Grassland (Table 2), which agrees with many enrichment and pure culture studies (Lehtovirta-Morley, 2018). Despite the overlap of substrate affinity between some oligotrophic AOB and non-marine AOA (Kits et al., 2017), the different K_m_’s between AOA and AOB have been generally considered a factor leading to niche differentiation (Martens-Habbena et al., 2009). Compared with AOB, the lower *K_m_* of AOA seem to reflect their smaller genome and cell volume, lower specific cell activities, and lower maintenance energy, which may have provided evolutionary advantages for AOA to thrive in nutrient-depleted environments (Könneke et al., 2005; Tourna et al., 2011; Urakawa et al., 2011; Hatzenpichler, 2012). While we do not have direct evidence, we suspect the bacterial nitrification inhibition by root exudates of *Bromus* spp. (O'Sullivan et al., 2017), a dominant species in our Grassland system, have resulted in the very low *K_m_* of AOB observed in the Grassland soils.

### Niche Differentiation Between AOA and AOB Induced by High NH_4_^+^ Inputs

To test the hypothesis of niche differentiation between AOA and AOB as a result of NH_4_^+^ inhibition, we first confirmed that nitrification potentials of AOA and AOB were stable across seasons, as noted above. Here for five out of eight ecosystems (including fertilized subplots), Haldane models provided significantly better fits than Michaelis-Menten models for AOB-derived nitrification kinetics (Figure 2, *p* < 0.05). In contrast, AOA-derived nitrification exhibited Michaelis-Menten kinetics in all ecosystems but one (Deciduous forest).

This suggests that AOA are more resistant to high N inputs than are AOB, which challenges the conventional view, based primarily on marine AOA strains, that AOA are less tolerant to high NH_4_^+^ concentrations (Könneke et al., 2005; Martens-Habbena et al., 2009). Consistent with our findings, Lehtovirta-Morley et al. (2016) discovered a soil AOA isolate capable of tolerating more than 100 mM of NH_4_^+^, which is higher than that for even most AOB strains, typically inhibited at 7–50 mM NH_4_^+^ (Prosser and Nicol, 2012). While the mechanism for higher NH_4_^+^ tolerance of AOA than AOB is not entirely clear, it is possible that the *Nitrosocosmicus franklandus*-like AOA are numerically prevalent or even functionally dominant in our ecosystems, although this hypothesis merits further genomic sequencing analysis. Together, these results suggest that in addition to substrate affinity, soil pH, and mixotrophy as reviewed by Prosser and Nicol (2012), NH_4_^+^ inhibition of AOB relative to AOA may be another important factor leading to niche differentiation between AOA and AOB in soils.

### Soil pH as a Predictor of *V_max_*

Existing studies typically examined correlations between potential nitrification rates and *amoA* copy numbers for AOA and AOB (He et al., 2007; Shen et al., 2008; Bernhard et al., 2010; Zeglin et al., 2011; Lu et al., 2015; Ouyang et al., 2016). However, no theoretical basis has been proposed to support a similar response of nitrifier abundance and activities to environmental disturbance. In addition, *amoA* gene abundance may not be a reliable surrogate for nitrification activities (Prosser and Nicol, 2012). It is well known that the presence of functional genes does not necessarily indicate active microbial communities (Levy-Booth et al., 2014; Bier et al., 2015). Not surprisingly, no significant correlations between *V_max_* and *amoA* copy numbers across a management gradient were found in this study for either AOA or AOB (Figure 3D).

Consistent with our fourth hypothesis, results show soil pH is a strong and positive environmental factor for explaining maximum nitrification rate (*V_max_*) for both AOA and AOB (Figure 3C). Soil pH has been shown to be a strong selection force for shaping community composition of soil nitrifiers (Nicol et al., 2008; Hu et al., 2013; Baolan et al., 2014; Stempfhuber et al., 2015), although its effects on nitrification potentials are inconsistent: some acidic forest soils exhibit high nitrification potentials (Robertson and Vitousek, 1981; Robertson, 1982). Changes of community compositions could nevertheless affect maximum nitrification rates because *V_max_* measures the average nitrification of active nitrifiers under optimal conditions (Prosser and Nicol, 2012).

Results also show that nitrification activities of AOB were more positively influenced by soil pH than were AOA (28.5 vs. 13.1%, Figure 3C). This difference seems to coincide with the different pH optima reported for AOB and AOA, both in pure culture and in terrestrial environments. While AOB cultures typically cannot grow below pH 6.5 (Allison and Prosser, 1993; Jiang and Bakken, 1999), AOA appear to tolerate from very acidic (pH = 2.5) to alkaline (pH = 8–9) conditions (Erguder et al., 2009). Additionally, in acidic soils where AOA have been found to actively dominate nitrification (Gubry-Rangin et al., 2010; Jiang et al., 2015; Li et al., 2019b), AOB were more often in low abundance or even absent (Stopnišek et al., 2010; Yao et al., 2011).

### Implications for Modeling and Soil Nitrification Management

Overall, results show that while AOB nitrification can be inhibited by high NH_4_^+^ inputs and exhibit Haldane kinetics, AOA nitrification better fit Michaelis-Menten kinetics in general. Thus, results reveal the need for careful model selection in future kinetics and biogeochemical modeling studies that now only consider Michaelis-Menten models (Malhi and McGill, 1982; Müller et al., 2007; Palmer et al., 2012; Nowka et al., 2015).

Results also reveal the discrepancy between nitrification potential vs. nitrification kinetics assays. Despite the positive association between potential nitrification rates and *V_max_* observed among different ecosystems, the nitrification potential assays exhibited limitations when nitrification was inhibited by high NH_4_^+^ addition. Indeed, because of the phylogenetic diversity of soil nitrifiers and the subsequent variation in substrate affinity and NH_3_ tolerance, it seems impossible to generate one single optimal experimental condition that allows all nitrifiers to perform actively at the maximum rate (Hazard et al., 2020). Thus, future research using nitrification potential assays should be very careful about selecting concentrations of NH_4_^+^ additions. That said, although more labor-intensive, nitrification kinetic assay deserves to be considered if the goal is to measure the maximum nitrification rate.

In addition, since AOB generally have higher nitrification potentials and *V_max_* than AOA, the inhibition of AOB by high NH_4_^+^ could be a potential strategy for reducing N_2_O emissions. For example, Deppe et al. (2017) used a controlled uptake long-term ammonium nutrition (CULTAN) fertilization strategy to substantially reduce soil nitrification rates and concurrent N_2_O emissions by creating fertilizer microsites with extremely high NH_4_^+^ concentrations (>2000 mg N kg^−1^ soil, equivalent to >15 mM NH_4_^+^) but it is unclear if AOB rather than AOA were inhibited. Although eventually nitrifier inhibition will be relieved by dilution of NH_4_^+^ from plant uptake, precipitation, and diffusion away from microsites (Deppe et al., 2016), nevertheless future studies might explore the inhibition of AOB vs. AOA in soil NH_4_^+^ “hotspots” created by certain fertilizer placement techniques such as injection of anhydrous NH_3_ or fertilizer banding, and resulting N_2_O suppression.

## Data Availability Statement

Data used in this study will be made available at: https://doi.org/10.5061/dryad.573n5tb5w.

## Author Contributions

DL and GPR conceived and designed the study, interpreted the analyses, and wrote the paper with input from all contributors. DL conducted the study with molecular biology expertise from YO and LT and performed data analysis. All authors contributed to the article and approved the submitted version.

### Conflict of Interest

The authors declare that the research was conducted in the absence of any commercial or financial relationships that could be construed as a potential conflict of interest.

## Footnotes

^1^https://lter.kbs.msu.edu/datatables/237

^2^https://lter.kbs.msu.edu/datatables/237

^3^https://lter.kbs.msu.edu/datatables/238
