# Supplementary material for: Niche Differentiation of Bacterial Versus Archaeal Soil Nitrifiers Induced by Ammonium Inhibition Along a Management Gradient
Source: Front Microbiol. 2020 Nov 12;11:568588. doi: 10.3389/fmicb.2020.568588 (PMC7689314; doi:10.3389/fmicb.2020.568588)
Supplement: Supplementary file 1 [file Data_Sheet_1.pdf]

Supplementary Material

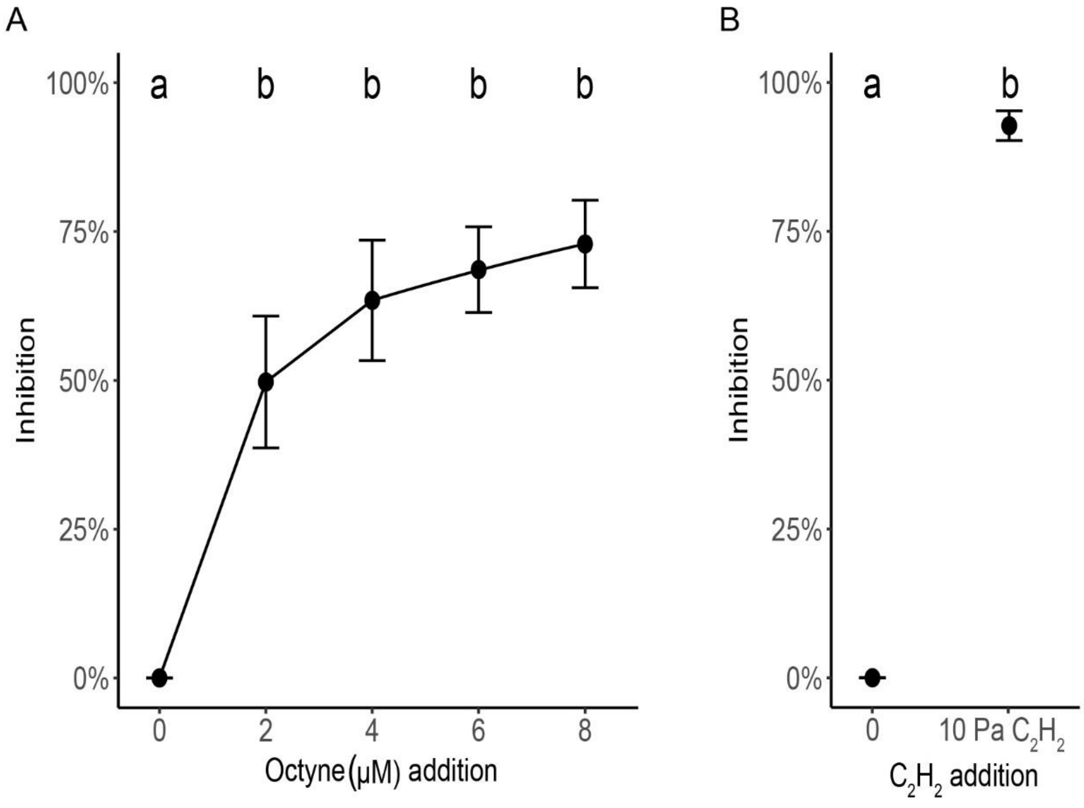

Supplementary Figure 1. Inhibition of AOB-derived NO_2^- + NO_3^- production by (A) octyne and (B) 10 Pa C_2H_2 in all systems. Bars represent standard errors (n=6 ecosystems: Conventional, Biologically-based, Poplar, Early successional, Grassland and Deciduous forest systems). Inhibition (%) is calculated as the percentage reduction of accumulated NO_2^- + NO_3^- compared with no inhibitor added. Different lowercase letters indicate significantly different inhibition effects among soils treated with (A) different amount of octyne and (B) 10 Pa C_2H_2 ($P < 0.05$).
