# Supplementary material for: Niche Differentiation of Bacterial Versus Archaeal Soil Nitrifiers Induced by Ammonium Inhibition Along a Management Gradient
Source: Front Microbiol. 2020 Nov 12;11:568588. doi: 10.3389/fmicb.2020.568588 (PMC7689314; doi:10.3389/fmicb.2020.568588)
Supplement: Supplementary file 2 [file Data_Sheet_2.docx]

Supplementary Material

**Supplementary Table 1**. Mean K_m_ and K_i_ (based on NH_3_) of managed and unmanaged systems for AOA and AOB; “+N” indicates subplots receiving long-term N fertilizer. Parameters are estimated with soil pH and temperature (25 °C) from Table 2. For each ecosystem, a “–” indicates Michaelis-Menten model is applied and thus K_i_ does not exist. See Emerson et al (1975) for details of converting NH_4_^+^ into NH_3_.

|  |  | | AOA | | AOB | |
| --- | --- | --- | --- | --- | --- | --- |
|  |  |  | K_m_ (nM) | K_i_ (µM) | K_m_ (nM) | K_i_ (µM) |
| Managed | Conventional | | 17.8 | – | 71.7 | – |
|  | Biologically-based | | 8.38 | – | 27.1 | 154 |
|  | Poplar | | 2.87 | – | 8.76 | 18.8 |
| Unmanaged | Early successional | 0N | 0.03 | – | 6.42 | – |
|  |  | +N | 3.82 | – | 8.99 | 9.87 |
|  | Deciduous forest | 0N | 0.03 | 2.90 | 0.27 | 5.68 |
|  |  | +N | 0.02 | – | 1.70 | 0.10 |

**Reference**

Emerson, K., Russo, R.C., Lund, R.E., and Thurston, R.V. (1975). Aqueous Ammonia Equilibrium Calculations: Effect of pH and Temperature. *J Fish Res Board Can* 32, 2379-2383. doi: 10.1139/f75-274.
